# The impact of loneliness and social isolation during COVID-19 on cognition in older adults: a scoping review

**DOI:** 10.3389/fpsyt.2023.1287391

**Published:** 2023-11-16

**Authors:** Kareena Kassam, Jacqueline M. McMillan

**Affiliations:** ^1^Mount Royal University, Calgary, AB, Canada; ^2^Division of Geriatric Medicine, Department of Medicine, University of Calgary, Calgary, AB, Canada; ^3^O’Brien Institute for Public Health, University of Calgary, Calgary, AB, Canada

**Keywords:** COVID-19, SARS CoV-2, loneliness, social isolation, cognition, cognitive decline, older adults

## Abstract

**Background:**

The COVID-19 pandemic required implementation of public health measures to reduce the spread of SARS CoV-2. This resulted in social isolation and loneliness for many older adults. Loneliness and social isolation are associated with cognitive decline, however, the impact of this during COVID-19 has not been fully characterized.

**Objective:**

The aim of this scoping review was to explore the impact of social isolation and loneliness during COVID-19 on cognition in older adults.

**Eligibility criteria:**

Eligible studies occurred during the COVID-19 pandemic, enrolled older adults and reported longitudinal quantitative data on both loneliness (exposure) and cognition (outcome).

**Sources of evidence:**

A comprehensive search was conducted in CINAHL, Medline, PubMed, and Psychinfo databases (updated October 10, 2023).

**Charting methods:**

Studies were screened independently by two reviewers and study characteristics, including participant demographics, loneliness and cognition measurement tools, study objectives, methods and results were extracted.

**Results:**

The search yielded 415 results, and seven were included in the final data synthesis. All studies were conducted between 2019 and 2023. Six studies enrolled community-dwelling individuals while the remaining study was conducted in long-term care. In 6 studies, loneliness and/or social isolation was correlated with poorer cognitive function. In the seventh study, subjective memory worsened, while objective cognitive testing did not.

**Conclusion:**

Loneliness and social isolation during COVID-19 were correlated with cognitive decline in older adults. The long-term effect of these impacts remains to be shown. Future studies may focus on interventions to mitigate the effects of loneliness and social isolation during future pandemics.

## Introduction

The Corona Virus Disease 2019 (COVID-19) pandemic has had a significant impact on global health, leading to widespread adoption of public health measures to reduce the spread of SARS CoV-2 (1). For some older adults, these measures led to social isolation and loneliness (2, 3). Older adults are at particular risk of social isolation and loneliness for reasons including retirement, limited mobility, and/or death of a loved one (4). Social isolation and loneliness are associated with adverse cognitive outcomes, including dementia (5–15). It is crucial, therefore, to explore the implications of pandemic restrictions on the cognitive health of older adults, as it relates to social isolation and loneliness.

Loneliness is defined as the subjective feeling of being socially disconnected or lacking social connections (16). It has been identified as a significant public health issue (17). Loneliness is associated with negative health outcomes, including hypertension (18) cardiovascular disease, stroke (19, 20) and cognitive decline (8–15). Social isolation can lead to depression and cognitive inactivity which are linked to cognitive impairment (2, 5–7, 14). However, the impact of social isolation and loneliness on cognitive health during the COVID-19 pandemic, remains relatively unexplored. Maintaining social connections and relationships are essential to preserving cognitive function in older adults (21). The COVID-19 pandemic introduced new challenges to social connection including disruption of regular activities, limited social gatherings, and reduced visitation in hospitals (22, 23). Social isolation and loneliness during the pandemic may increase subjective and objective cognitive concerns, and further cognitive decline in those with pre-existing dementia (24). Exploring the relationship between social isolation/loneliness and cognitive decline in the context of the COVID-19 pandemic will provide insight into depth of the problem as well as the potential interventions to combat this issue.

The aim of this scoping review is to address the gap in knowledge by synthesizing the available evidence on the impact of loneliness and social isolation during the COVID-19 pandemic on cognition in older adults. The scoping review approach permits a broad and exploratory evaluation of the current literature. This review aims to provide a comprehensive analysis of the relationship between these variables by examining longitudinal studies that quantitatively measured both loneliness and cognitive function at two times points during the COVID-19 pandemic. The findings will inform healthcare professionals and policy makers of the degree of the impact of loneliness and social isolation on cognitive function during the pandemic.

## Methods

The protocol for this scoping review was developed in accordance with the PRISMA-ScR (Preferred reporting items for systematic reviews and meta-analyses extension for scoping reviews) (25) (Supplementary Table S1).

### Search strategy

With the assistance of a research librarian, the following search terms were utilized: “loneliness,” “social isolation,” “COVID-19,” “coronavirus,” “SARS-COV-2,” “older adults” “aged,” “frail elderly,” “cognition,” “cognitive decline,” “mild cognitive impairment,” “dementia” and “cognitive dysfunction.” The search was run on April 26, 2022 using the following databases: CINAHL, Medline, PubMed, and Psych info. The search was updated using the same search terms and same databases on October 10, 2023.

### Eligibility criteria

The search results were exported into Covidence,^1^ where duplicates were removed. Titles and abstracts were screened by two independent reviewers to determine eligibility for full text review. Articles were considered eligible if the study occurred during the COVID-19 pandemic, enrolled older adults (≥ 50 years), and reported a quantitative measure of both loneliness (exposure) and cognition (outcome). Only studies with longitudinal data were included. Only studies that investigated the association between measures of loneliness/social isolation and cognitive outcome were included. Exclusion criteria: non-English language, age < 50 years, studies conducted prior to the COVID-19 pandemic, no quantitative measure of loneliness and/or cognition, cross-sectional study, case–control, case-report, reviews and editorials were excluded.

### Data extraction

Data were extracted and entered into an Excel template. One author independently extracted data for half of the studies, while another author extracted data for the other half of the studies. Each author independently reviewed the data extraction of the other author to confirm accuracy. The following data were extracted: author, year, country, setting, number of participants, demographics (mean or median age of participants and percentage female), presence (or absence) of dementia at baseline, study start and end dates (to confirm occurrence during the COVID-19 pandemic), loneliness measurement tool used, cognition measurement tool used, study objective(s), methods, and results. Any discrepancies were resolved by consensus.

## Results

**Figure 1:**
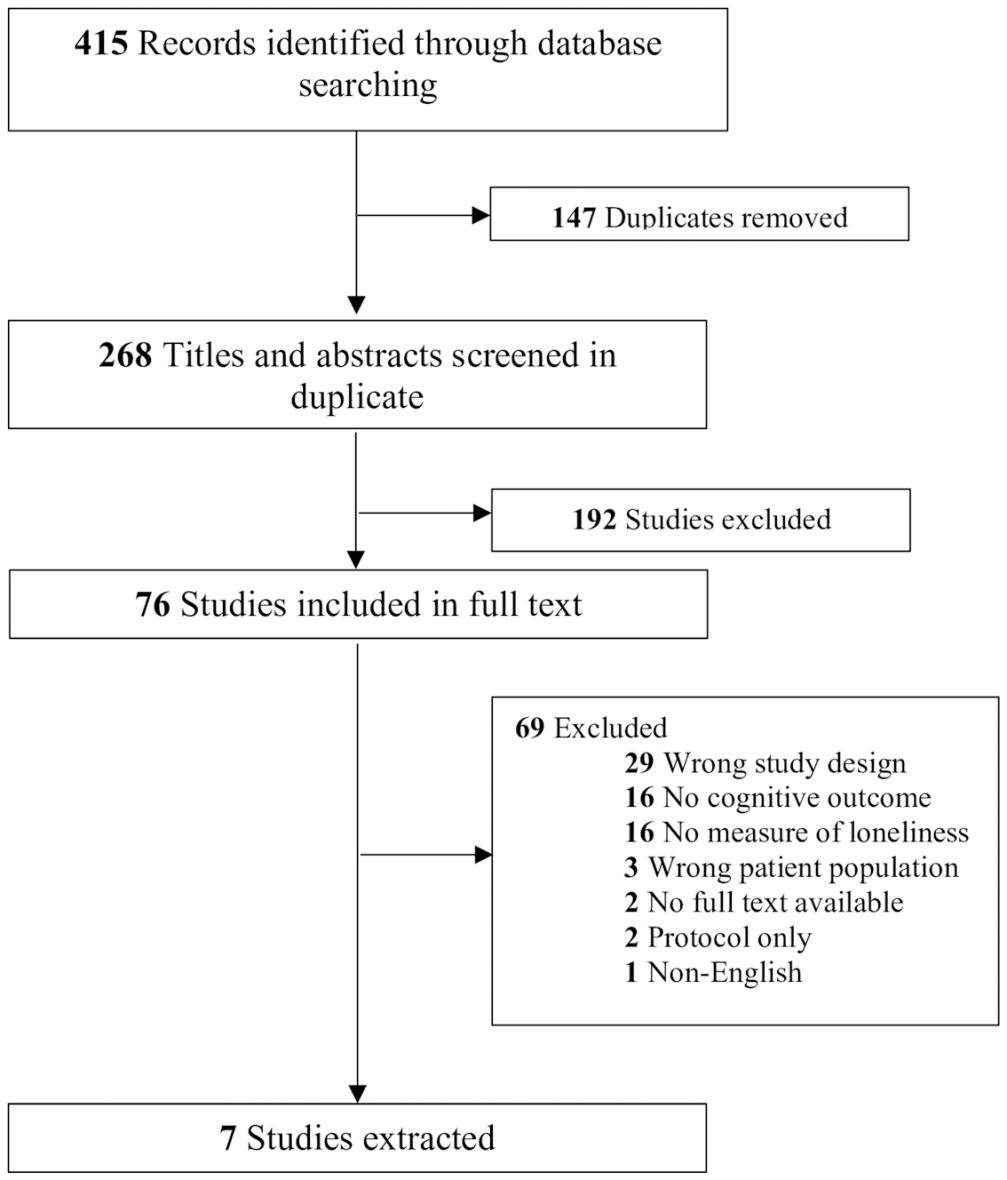
PRISMA flow diagram.

The search yielded 415 results (270 from original search-April 26, 2022 and 145 from the updated search-October 10, 2023) These are summarized in the PRISMA flow diagram (Figure 1). Details of the search strategy are found in Supplementary Table S2. One-hundred and forty-seven duplicates were removed (95 from the original search-April 2022 and 52 from the updated search-October 2023) leaving 268 unique titles and abstracts which were reviewed independently to determine eligibility for inclusion (175 from the original search-April 2022 and 93 from the updated search-October 2023). One-hundred and ninety-two studies were excluded after title and abstract screening (109 from the original search and 83 from the updated search). The most common reasons for exclusion were: no longitudinal date (i.e., wrong study design), no cognitive outcome, no measure of loneliness, or no older adult population (i.e., wrong patient population). Seventy-six studies were included in full-text review (66 from the original search and 10 from the updated search), again, completed in duplicate by two independent reviewers. Sixty-nine citations (60 from the original search and 9 from the updated search) were excluded after full text review. The most common reasons for exclusion were: wrong study design/no longitudinal data (*n* = 29), no cognitive outcome (*n* = 16), no measure of loneliness (*n* = 16), no older adult population (*n* = 3), no full text available (*n* = 2), protocol only (*n* = 2), and non-English language (*n* = 1). Seven studies met inclusion criteria and were included in the final data synthesis (6 from the original search and 1 from the updated search).

All studies were conducted between 2019 and 2023 (Table 1 Characteristics of Included Studies). Studies were conducted in Korea, China, Spain, the United States of America, Japan, Portugal, and Scotland. Six studies enrolled community-dwelling individuals and one was conducted in a long-term care facility and enrolled residents of the facility. Study sizes ranged from 98 to 2,792 participants and the mean age of participants ranged from 68.2 to 84 years. The proportion of female participants ranged from 54.7 to 74.7%. Three studies enrolled participants who were living with dementia or cognitive impairment at baseline (Figure 1).

**Table 1:** Characteristics of included studies.

Author, year (country)	Setting	Time period	Sample size	Mean age (years)	Percentage female	Participant characteristics	Social isolation or loneliness measure	Cognition measure	Outcomes
Chen et al. (26) (China)	Community	September 2019–September 2020	177	MCI 68.7; AD 71.5; DLB 74	MCI 57.9%; AD 58%; DLB 45.4%	All had some form of cognitive impairment at baseline	Self-rated questionnaire that assessed the number and frequency of contacts with relatives and friends	MMSE; MoCA	In AD patients, MMSE declined by 1.6 and MoCA declined by 1.0 at one-year follow-up. In DLB patients, MMSE declined by 3.6 and MoCA declined by 2.5 at one-year follow-up. Decline in social contact was associated with decline in MMSE scores in DLB patients.
Kobayashi et al. (27) (United States)	Community	April 2020–May 2021	2204	68.2, 95% CI 67.5, 68.8	58.2%	No known dementia at baseline	3-item UCLA Loneliness Scale	PROMIS; Cognitive Function and Abilities Scale	Over a nine-month period (August 2020 to May 2021), both between-person and within-person change in loneliness was negatively associated with perceived cognitive function and abilities.
Noguchi et al. (28) (Japan)	Community	March 2020; October 2020	955	79.6 SD 4.7	54.7%	No dementia at baseline	Social Isolation Index. Total scores range from 0 to 5 with higher scores indicating greater social isolation. Scores of ≥3 were defined as social isolation	Self-reported Cognitive Performance Scale. Four item scale resulting in hierarchical 7-category scale ranging from 0 to 6 with higher scores indicating greater impairment.	504 (52.8%) remained non-isolated 46 (4.8%) became isolated from nonisolation 67 (7.0%) became nonisolated from isolation 98 (10.3%) were consistently isolation Cognitive decline occurred in 54 (5.7%) Compared to nonisolated, OR for cognitive impairment for isolated from nonisolation was 2.74 (95% CI 1.13, 6.61) and OR for consistent isolation was 2.33 (95% CI 1.07, 5.05)
Nogueira et al. (28) (Portugal)	Community	NR	150	69.02 SD 7.95	74.7%	No dementia at baseline	Lubben Social Network Scale; UCLA Loneliness Scale	MMSE; MOCA; Trail-making test A/B; digit-symbol coding; digit span; fluencies protocol Cognitive Decline Complaints Scale	There were no correlations found between social isolation or loneliness and cognitive decline based on objective measures of cognition (i.e. MMSE, MoCA, TMT, DSC, DS and FP), despite participants’ report of worsened subjective cognition
Okely et al. (29) (Scotland)	Community	May 2020–June 2020	137	84	NR	NR	Single item question about loneliness. Responses categorized 1 to 4 with higher scores indicating greater lonliness. 7-item social support scale. Scores of 0 to 14 with higher scores indicating greater social support.	Self-reported responses to 5 questions about memory. Scored 0 to 5 with higher scores indicating more severe subjective memory impairment.	The study found that decreased social support was associated with an increase in self-reported memory problems (r = −0.169; *p* < 0.05)
Pereiro et al. (30) (Spain)	Long-term care	July 2020–September 2020	98	83.41 SD 9.61	62%	68 participants (69%) had a CDR score of 1,2,or 3 (mild, moderate or severe cognitive impairment) Baseline CDR score 0 = 10 participants CDR 0.5 = 20 participants CDR 1 = 19 participants CDR 2 = 23 participants CDR 3 = 26 participants	Social contact frequency 1 = without contact 2 = biweekly/monthly 3 = weekly 4 = daily	MMSE; Clinical Dementia Rating Scale	MMSE scores were significantly lower in the third pre-lockdown period and the post-lockdown period, and the scores were lower in persons in the mild, moderate and severe CDR groups, than in the normal or questionable CDR groups. When frequency of social contact was analyzed as a covariate, the differences in MMSE scores was eliminated
Lee and Kim, (31)	Community	July 1016–September 2022	2792	69.3 before pandemic, 71.4 after pandemic	57.9% before pandemic, 58.4% after pandemic	NR	Social Connectedness Single survey question: “How often do you meet friends or relatives in person?”	Korean MMSE	An increase in one unit in the frequency of contact with familiar individuals increased cognitive scores by 0.1470 (SE 0.0677; *p* < 0.01)

**Footnote**. **MCI** Mild Cognitive Impairment; **AD** Alzheimer Dementia; **DLB** Dementia with Lewy Bodies; **CI** Confidence Interval; **SD** Standard deviation; **MMSE** Mini Mental State Examination; **MoCA** Montreal Cognitive Assessment; **NPI** Neuropsychiatric Inventory; **CPS** Cognitive Performance Scale; **UCLA** University of California Los Angeles; **PROMIS** Patient-Reported Outcomes Measurement Information System; **NR** not reported; **TMT** Trail-making test; **DSC** digit symbol coding; **DS** digit span; **FP** fluencies protocol; **CDCS** Cognitive Decline Complaints Scale; **CDR** Clinical Dementia Rating; **SE** standard error.

Loneliness was measured using the 3-item UCLA Loneliness scale in 2 studies, a self-rated questionnaires in 5 studies, social connectedness in 1 study and the Lubben Social Network Scale in 1 study. One study used more than one loneliness and/or social isolation measurement scale.

Cognition was measured using the Mini Mental State Exam (MMSE) in 4 studies, Montreal Cognitive Assessment (MoCA) in 2 studies, Cognitive Performance Scale (CPS) in 1 study, Patient Reported Outcomes Measurement Information System (PROMIS) in 1 study, and Clinical Dementia Rating (CDR) in 1 study. A self-reported questionnaire was used in 1 study and the Trail Making Test (TMT), digit-symbol coding (DSC), digit span (DS) and fluencies protocol (FP) were all used in a single study. Five studies used more than one cognitive measurement scale.

### Summary of findings

#### Community-dwelling individuals without known pre-existing cognitive impairment

Recognizing that the relationship between social connectedness and cognitive function is likely bidirectional the authors of the most recently published study (31) used the COVID-19 pandemic as an instrumental variable to estimate the causal effect of social connectedness on cognitive functions. This approach uses two-stage least squares regression to overcome potential omitted variables bias or reverse causality. They found that participants, on average, reduced the number of meetings per month with family and friends from 5.54 pre-pandemic to 4.54 during the pandemic. Cognitive scores also decreased during the pandemic by an average of 0.3131 on the Korean MMSE. However, the authors determined that an average one unit increase in participants’ frequency of meeting with family and friends was associated with an increase in cognitive scores on the Korean MMSE of 0.1470 (standard error 0.0677; *p* < 0.01). The authors concluded that cognitive scores decreased during the COVID-19 pandemic, but that increased social connectedness was associated with increased cognitive scores.

The United States COVID-19 Coping Study (27) investigated between-person and within-person differences in loneliness and self-reported cognitive function and abilities (27). A total of 2,204 middle-aged and older adults (≥55 years) were enrolled (27). Loneliness was measured using the 3-Item UCLA Loneliness Scale with scores ranging from 3 to 9 (higher scores indicating greater loneliness) (32). Cognitive outcomes were assessed with the 6-item Patient Reported Outcomes Measurement Information System (PROMIS) Cognitive Function and Abilities Scales (33). Exposure and outcomes were assessed at four-months (August/September 2020; analytic baseline), six-months (October/November 2020), eight-months (December 2020/January 2021), ten-months (February/March 2021), and twelve-months (April/May 2021) (27). Between-person models were used to describe the mean change in the cognitive T-score in any month in which the loneliness score was one-unit greater than the sample mean. Within-person models described the mean change in the cognitive T-score in any month in which the loneliness score was one-unit greater than the individual’s personal mean. Increased loneliness compared to other adults, and to an individuals’ usual level, were associated with worse self-reported cognitive function and ability. Between-person differences in loneliness scores were negatively associated with cognitive function and abilities [*β* = −1.01 (−1.43, −0.59) and *β* = −0.95 (−1.10, −0.72, respectively), respectively]. Similarly, within-person changes in loneliness were negatively associated with cognitive function and abilities [β = −0.83 (−1.40, −0.26) and *β* = −0.74 (−1.10, −0.38), respectively].

Noguchi et al. investigated the association between social isolation and cognitive decline during COVID-19 in Japan (28). Participants were adults ≥65 years randomly sampled from the community (28). A baseline survey was conducted between March 3–16, 2020 (before the emergency declaration) (28). A follow up survey was conducted between October 16–30, 2020 (after the emergency declaration) (28). Individuals with cognitive impairment at baseline were excluded (28). Based on the individuals’ social isolation index scores at baseline and follow-up, participants were divided into four groups: “remained nonisolated” (*n* = 504; 52.8%), “isolated from nonisolation” (*n* = 46; 4.8%), “non-isolated from isolation” (*n* = 67; 7.0), and “consistent isolation” (*n* = 98; 10.3%). Fifty-four individuals (5.7%) developed cognitive impairment at the time of follow-up (28). Compared with the group that remained non-isolated, the isolated from nonisolated and consistent isolation groups were significantly associated with cognitive impairment at follow-up (28). The odds ratios for cognitive impairment at follow-up were 2.74 [95% Confidence Interval (CI) 1.13–6.61; *p* = 0.026] for isolated from non-isolation, and 2.33 (95% CI 1.07–5.05, *p* = 0.033) for consistent isolation (28).

Nogueira et al. (34) enrolled 150 cognitively healthy older Portugese adults based on the following inclusion criteria: ≥50 years of age, at least 1 year of formal education, Portuguese speaking, and absence of psychiatric, neurological or psychological clinical condition (34). Participants were excluded if they had a clinical condition that affected cognitive performance, if they consumed any medication impacting cognitive function (the medications included were not defined) and/or if they possessed a significant functional incapacity (not defined) (34). A baseline assessment was completed before the COVID-19 pandemic and a follow-up was completed during the term of COVID-19 confinement (average 1 year and 5 months apart) (34). Baseline and follow-up assessments included: the MMSE, MoCA, Trail Making Test A/B, Digital Symbol Coding, Digit Span, and the Fluencies Protocol (34). The Cognitive Decline Complaints Scale (CDCS) was used to measure self-reported cognitive decline (34). To measure social network and loneliness, the Lubben Social Network Scale and UCLA-Loneliness Scale were used (34). Subjective memory significantly worsened based on Cognitive Decline Complaints Scale scores between the time of the 1st (21.75 ± 14.44) and 2nd assessment [24.49 ± 15.16; *t* (82) = −2.32, *p* = 0.023] (34). Additionally, UCLA-16 scores were significantly correlated with CDCS scores (*r* = 0.440; *p* < 0.001). For most of the objective cognitive tests, there were no significant differences in scores and no correlations were found between social isolation or loneliness and objective cognitive decline despite participants’ report of worsened subjective cognition (34).

Okely et al. enrolled 137 participants from the Lothian Birth Cohort 1936 study (29). Participants completed a questionnaire before the COVID-19 lockdown (T1, 2017–2019) and then again during the lockdown (T2; May 27–June 8, 2020) (29). The questionnaire assessed self-reported memory problems, loneliness, psychological wellbeing, social support, neighborhood cohesion, physical activity, and sleep quality (29). Memory problems were assessed using five “yes” or “no” questions. Responses were scored on a scale from 0 to 5 (higher scores reflecting more severe memory problems). Loneliness was measured with a single question: “How much of the time during the past week have you felt lonely”? Answers were scored on a scale from 1 to 4 (higher scores reflecting greater feelings of loneliness). The study found that decreased social support was correlated with an increase in self-reported memory problems (*r* = −0.169; *p* < 0.05) (29). The relationship between change in loneliness and change in subjective cognition was not evaluated.

#### Community-dwelling individuals with known pre-existing cognitive impairment

Chen et al. investigated the impact of the COVID-19 lockdown on cognition in older adults with mild cognitive impairment (MCI), Alzheimer’s disease (AD) and dementia with Lewy bodies (DLB) (26). Participants were assessed before the COVID-19 pandemic (September 20–December 31, 2019) and again at 1 year follow-up (October 21–November 30, 2020) Exclusion criteria included: severe loss of vision or hearing, physical disability, lost to follow-up, newly occurring delirium, stoke and/or life-threatening illness (26). Participants with MCI who subsequently developed dementia or who were misdiagnosed initially with the diagnosis corrected after being reassessed were excluded (26). At T2, social contact significantly decreased in all 3 groups. Cognitive function (MMSE and MoCA) did not significantly decline in MCI group, but in patients with AD and DLB, the MMSE, and MoCA worsened during the pandemic compared to pre-pandemic. MMSE scores declined by 3.6 points and MoCA scores by 2.5 points in the DLB patients between baseline and follow-up (1 year later) (26). In AD patients, MMSE scores declined by 1.6 and MoCA scores by 1.0 between baseline and follow-up. There was an association between decline in social contact and decline in MMSE scores in patients with DLB (*β* = 0.491; *p* = 0.034), and between decline in social contact and worsening neuropsychiatric inventory scores in both AD (*β* = 0.204; *p* = 0.045) and DLB (*β* = 0.552; *p* = 0.013) patients at 1 year (26).

#### Residents in long-term care

Pereiro et al. investigated the effect of the COVID-19 lockdown on cognitive decline in a care facility in Spain (30). Inclusion criteria were: residing at the care facility for the entire period of confinement (July 2020–September 2020), ≥60 years of age, and completion of three assessments prior to the lockdown (30). Cognitive assessments included the Spanish MMSE and Clinical Dementia Rating (CDR); social contact frequency was determined by staff who answered the question, “the person was able to communicate with their family and friends by phone or other telematic means” using the scale: 1 = without contact; 2 = biweekly/monthly; 3 = weekly; 4 = daily (30). Three pre-lockdown assessments were completed (30) and a follow-up assessment was completed post-lockdown (July–September 2020) (30). Ten percent of those with a baseline CDR score of zero (no cognitive impairment) had no contact and 30% had daily contact. In contrast, 50% of those with a baseline CDR score of 3 (severe cognitive impairment) had no contact and 0% had daily contact. MMSE scores were significantly lower in the third pre-lockdown period and the post-lockdown period, and the scores were lower in persons in the mild, moderate and severe CDR groups, than in the normal or questionable CDR groups (30). When social contact frequency was included as a covariate, the significance of the differences in the MMSE scores disappeared (30).

## Discussion

Older adults are more vulnerable to the negative outcomes of infection with SARS-CoV-2 (35). In order to reduce virus transmission, public health measures were instituted to prevent infection and adverse sequelae (23). Physical distancing lead to increased feelings of loneliness and social isolation for older adults, both of which are associated with cognitive decline in this population (5–15, 36). In the present scoping review, we investigated the impact of loneliness and social isolation during the COVID-19 pandemic on cognition in older adults. The present study is unique in focussing on the cognitive impacts of loneliness and social isolation that resulted from mandate rather than personal choice or circumstance. For a period of time, the required isolation was unrelenting and when paired with the fear and concern of contracting SARS-CoV2 infection, the two may have combined synergistically to create a profound negative impact on mental health. The majority of included studies reported that loneliness and social isolation were correlated with poorer cognition (26–29). In one study, although subjective memory concerns worsened during lockdown, objective cognitive testing in most cases did not (34). In this study, loneliness was significantly correlated with worsened subjective cognitive complaints (34). In the single study set in LTC, although MMSE worsened over time, when social contact was included as a covariate, the decline in MMSE was no longer significant (30).

Chen et al. measured a decline in MMSE from prior to the COVID-19 pandemic, to a period during the lockdown that was greater in persons with Dementia with Lewy Bodies, than persons with Alzheimer’s Dementia (MMSE decline of 3.6 in DLB versus 1.6 in AD). There was a similar, albeit smaller decline in MoCA scores between the two dementia subtypes, whereby persons with DLB experienced a decline in the MoCA score of 2.5 and persons with AD experienced a decline in the MoCA score of 1.0. The observation of a more rapid decline in MMSE scores in DLB compared to AD has been observed previously (37). In the earlier study, MMSE scores in persons with DLB declined by 2.1 per year and MMSE scores in persons with AD declined by 1.6 (37). Another study has also reported a shorter time to severe dementia in persons with DLB than in persons with AD (38).

In six of the 7 included studies, loneliness or social isolation was correlated with worsened cognition during the COVID-19 pandemic. Whether this effect will persist into the future and impact on dementia incidence remains to be determined. Additionally, the persistence of social isolation and loneliness which older adults may have experienced during periods of lockdown has yet to be fully evaluated. A growing body of evidence supports the association between social isolation and loneliness and an increase in the risk of all-cause and Alzheimer dementia (13, 14). Therefore, the observations that older adults experienced increased social isolation and loneliness as a result of lockdowns during COVID-19, combined with the short-term evidence provided here, demands a thoughtful review of the approach to protecting older adults during future pandemics. Social isolation has been proposed as a potentially modifiable risk for dementia in the Lancet Commissions (39, 40). As such, creative approaches to balancing the risk of infection while maintaining social engagement and connections for older adults who are at risk of or experiencing cognitive decline are needed. The involvement of patients and caregivers in future studies would provide a valuable perspective of the goals, values and preferences of this patient population. Fifty-percent of older adults increased their nonphysical intergenerational contacts during the COVID-19 lockdown, especially through the use of video calls, instant messages and social media use (41). Education and assistance with these types of communication may be an avenue of supporting older adults to remain socially connected during future pandemics.

## Conclusion

In this scoping review, loneliness and social isolation during with the COVID-19 pandemic lockdown were correlated with a decline in cognition in the older adult population. These consequences were seen in both community-dwelling individuals, as well as residents in LTC facilities, and in participants living with and without pre-existing cognitive decline. These results explore the depth and extent of the impact of loneliness and social isolation on cognition, and may be utilized to address this unintended consequence while minimizing viral transmission. Gaps that have been identified include effective interventions, as well as patient and caregiver perspectives. Future studies may focus on developing interventions to address and mitigate the effects of loneliness and social isolation during future global pandemics. The relationship between social isolation/loneliness and cognition with respect to the COVID-19 pandemic requires further research in order to determine appropriate interventions. Studies which enroll patient and caregiver partners are also warranted so that priorities may be established and patient perspectives are incorporated when future lockdown policies are made and enacted.

## Limitations

This study has limitations. Most studies enrolled community-dwelling older adults, providing limited generalizability of the results to residents of LTC facilities. Additionally, we included only longitudinal study designs in order to better evaluate the change in loneliness and cognition over time. Only English studies were included which may have resulted in an omission of studies evaluating the effect of loneliness on cognition that may have been published in a non-English language. Some studies employed self-reported cognitive measures which may be vulnerable to recall and social desirability bias. Lastly, it is possible that the mandated, and for a period of time, unrelenting requirement for isolation, may have led to different cognitive outcomes, than the cognitive outcomes that have been more broadly researched and result from individuals who become lonely and/or socially isolated due to choice or circumstance. Future studies are needed to determine whether there is a divergence of outcomes depending on these factors.

## Data availability statement

The original contributions presented in the study are included in the article/Supplementary material, further inquiries can be directed to the corresponding author/s.

## Author contributions

KK: Conceptualization, Data curation, Methodology, Writing – original draft. JM: Conceptualization, Data curation, Methodology, Supervision, Writing – review & editing.

## Footnotes

^1^ https://www.covidence.org/
