# Supplementary material for: The impact of loneliness and social isolation during COVID-19 on cognition in older adults: a scoping review
Source: Front Psychiatry. 2023 Nov 16;14:1287391. doi: 10.3389/fpsyt.2023.1287391 (PMC10690360; doi:10.3389/fpsyt.2023.1287391)
Supplement: Supplementary file 1 [file Table_1.pdf]

Table 1. Study Characteristics

**Author, year (country)**	**Setting**	**Time period**	**Sample size**	**Mean age (years)**	**Percentage Female**	**Participant characteristics**	**Social Isolation or Loneliness measure**	**Cognition measure**	**Outcomes**
Chen et al. 2021 (China)	Community	September 2019-September 2020	177	MCI 68.7; AD 71.5; DLB 74	*MCI 57.9%; AD 58%; DLB 45.4%*	All had some form of cognitive impairment at baseline	Self-rated questionnaire that assessed the number and frequency of contacts with relatives and friends	MMSE; MoCA	In AD patients, MMSE declined by 1.6 and MoCA declined by 1.0 at one-year follow-up. In DLB patients, MMSE declined by 3.6 and MoCA declined by 2.5 at one-year follow-up. Decline in social contact was associated with decline in MMSE scores in DLB patients.
Kobayashi et al. 2022 (US)	Community	April 2020-May 2021	2204	68.2, 95% CI 67.5, 68.8	*58.2%*	No known dementia at baseline	3-item UCLA Loneliness Scale	PROMIS; Cognitive Function and Abilities Scale	Over a nine-month period (August 2020 to May 2021), both between-person and within- person change in loneliness was negatively associated with perceived cognitive function and abilities.

Noguchi et al. 2021 (Japan)	Community	March 2020; October 2020	955	79.6 SD 4.7	*54.7%*	No dementia at baseline	Social Isolation Index. Total scores range from 0 to 5 with higher scores indicating greater social isolation. Scores of ≥3 were defined as social isolation	Self-reported Cognitive Performance Scale. Four item scale resulting in hierarchical 7-category scale ranging from 0 to 6 with higher scores indicating greater impairment.	504 (52.8%) remained non-isolated<br>46 (4.8%) became isolated from nonisolation<br>67 (7.0%) became nonisolated from isolation<br>98 (10.3%) were consistently isolation<br>Cognitive decline occurred in 54 (5.7%)<br>Compared to nonisolated, OR for cognitive impairment for isolated from nonisolation was 2.74 (95% CI 1.13, 6.61) and OR for consistent isolation was 2.33 (95% CI 1.07, 5.05)
Nogueira et al. 2021 (Portugal)	Community	NR	150	69.02 SD 7.95	*74.7%*	No dementia at baseline	Lubben Social Network Scale; UCLA Loneliness Scale	MMSE; MOCA; Trail-making test A/B; digit-symbol coding; digit span; fluencies protocol Cognitive Decline Complaints Scale	There were no correlations found between social isolation or loneliness and cognitive decline based on objective measures of cognition (i.e. MMSE, MoCA, TMT, DSC, DS and FP), despite participants' report of worsened subjective cognition

Okely et al. 2020 (Scotland)	Community	May 2020-June 2020	137	84	*NR*	NR	Single item question about loneliness. Responses categorized 1 to 4 with higher scores indicating greater lonliness. 7-item social support scale. Scores of 0 to 14 with higher scores indicating greater social support.	Self-reported responses to 5 questions about memory. Scored 0 to 5 with higher scores indicating more severe subjective memory impairment.	The study found that decreased social support was associated with an increase in self-reported memory problems (r = −0.169; p<0.05)
Pereiro et al. 2021 (Spain)	Long-term care	July 2020-September 2020	98	83.41 SD 9.61	*62%*	68 participants (69%) had a CDR score of 1,2,or 3 (mild, moderate or severe cognitive impairment) Baseline CDR score 0= 10 participants CDR 0.5= 20 participants CDR 1= 19 participants CDR 2= 23 participants CDR 3= 26 participants	Social contact frequency 1=without contact 2= biweekly/monthly 3= weekly 4=daily	MMSE; Clinical Dementia Rating Scale	MMSE scores were significantly lower in the third pre-lockdown period and the post-lockdown period, and the scores were lower in persons in the mild, moderate and severe CDR groups, than in the normal or questionable CDR groups. When frequency of social contact was analyzed as a covariate, the differences in MMSE scores was eliminated

Lee & Kim, 2023	Community	2016-2022	2792	69.3 before pandemic, 71.4 after pandemic	57.9% before pandemic, 58.4% after pandemic	NR	Social Connectedness Single survey question: “How often do you meet friends or relatives in person?”	Korean MMSE	An increase in one unit in the frequency of contact with familiar individuals increased cognitive scores by 0.1470 (SE 0.0677; p<0.01)

Footnote. **MCI** Mild Cognitive Impairment; **AD** Alzheimer Dementia; **DLB** Dementia with Lewy Bodies; **CI** Confidence Interval; **SD** Standard deviation; **MMSE** Mini Mental State Examination; **MoCA** Montreal Cognitive Assessment; **NPI** Neuropsychiatric Inventory; **CPS** Cognitive Performance Scale; **UCLA** University of California Los Angeles; **PROMIS** Patient-Reported Outcomes Measurement Information System; **NR** not reported; **TMT** Trail-making test; **DSC** digit symbol coding; **DS** digit span; **FP** fluencies protocol; **CDCS** Cognitive Decline Complaints Scale; **CDR** Clinical Dementia Rating; **SE** standard error
