# Supplementary material for: The impact of loneliness and social isolation during COVID-19 on cognition in older adults: a scoping review
Source: Front Psychiatry. 2023 Nov 16;14:1287391. doi: 10.3389/fpsyt.2023.1287391 (PMC10690360; doi:10.3389/fpsyt.2023.1287391)
Supplement: Supplementary file 2 [file Table_2.pdf]

Supplementary Table 2. Search Strategy with for Each Corresponding Database

**Database**	**Concept 1: older adult**	**Concept 2: social isolation OR loneliness**	**Concept 3: cognition**	**Concept 4: COVID-19**
CINAHL MeSH terms	MH “aged” OR MH “frail elderly” OR MH “aged” OR MH “geriatrics”	MH “social isolation” OR MH “loneliness”	MH “cognition” OR MH “dementia” OR MH “mild cognitive impairment”	MH “COVID-19” OR MH “SARS-CoV-2” OR MH “COVID-19 Pandemic”
OVID/Medline MeSH terms	aged/ OR frail elderly/	social isolation/ OR loneliness/	dementia/ OR cognition disorders/ OR cognitive dysfunction/	COVID-19/
PubMed	aged/ OR frail elderly/	social isolation/ OR loneliness/	cognition/ OR cognitive disorders/	COVID-19/
PsychINFO	geriatrics/ OR aging/ OR older adulthood/	social isolation/ OR social deprivation/ OR patient seclusion/ OR social connectedness/ OR social withdrawal/ OR social exclusion	cognitive impairment/ OR cognitive development/ OR cognitive aging/ OR cognitive ability/ OR cognition/	COVID-19/
Search terms used for all databases	older adult OR elderly OR senior OR geriatrics	loneliness OR social isolation	cognitive decline/ OR cognitive impairment/ OR cognitive ability OR cognitive performance	COVID-19 OR coronavirus OR sars-cov-2
